# Machine-Learning-Based Genome-Wide Association Studies for Uncovering QTL Underlying Soybean Yield and Its Components

**DOI:** 10.3390/ijms23105538

**Published:** 2022-05-16

**Authors:** Mohsen Yoosefzadeh-Najafabadi, Milad Eskandari, Sepideh Torabi, Davoud Torkamaneh, Dan Tulpan, Istvan Rajcan

**Affiliations:** 1 Department of Plant Agriculture, University of Guelph, Guelph, ON N1G 2W1, Canada; myoosefz@uoguelph.ca (M.Y.-N.); storabi@uoguelph.ca (S.T.); irajcan@uoguelph.ca (I.R.); 2 Département de Phytologie, Université Laval, Québec City, QC G1V 0A6, Canada; davoud.torkamaneh.1@ulaval.ca; 3 Department of Animal Biosciences, University of Guelph, Guelph, ON N1G 2W1, Canada; dtulpan@uoguelph.ca

**Keywords:** data-driven models, FarmCPU, genome-wide association study, MLM, QTL, soybean breeding, support-vector machine

## Abstract

A genome-wide association study (GWAS) is currently one of the most recommended approaches for discovering marker-trait associations (MTAs) for complex traits in plant species. Insufficient statistical power is a limiting factor, especially in narrow genetic basis species, that conventional GWAS methods are suffering from. Using sophisticated mathematical methods such as machine learning (ML) algorithms may address this issue and advance the implication of this valuable genetic method in applied plant-breeding programs. In this study, we evaluated the potential use of two ML algorithms, support-vector machine (SVR) and random forest (RF), in a GWAS and compared them with two conventional methods of mixed linear models (MLM) and fixed and random model circulating probability unification (FarmCPU), for identifying MTAs for soybean-yield components. In this study, important soybean-yield component traits, including the number of reproductive nodes (RNP), non-reproductive nodes (NRNP), total nodes (NP), and total pods (PP) per plant along with yield and maturity, were assessed using a panel of 227 soybean genotypes evaluated at two locations over two years (four environments). Using the SVR-mediated GWAS method, we were able to discover MTAs colocalized with previously reported quantitative trait loci (QTL) with potential causal effects on the target traits, supported by the functional annotation of candidate gene analyses. This study demonstrated the potential benefit of using sophisticated mathematical approaches, such as SVR, in a GWAS to complement conventional GWAS methods for identifying MTAs that can improve the efficiency of genomic-based soybean-breeding programs.

## 1. Introduction

Soybean (*Glycine max* [L.] Merr.) is known as one of the most important legume crops worldwide with substantial economic value [1]. Despite the importance of genetic improvements in soybean yield, the germplasm has, in general, a narrow genetic basis, especially within North America, which has resulted in the limited progress of genetic gains for seed yield [2]. Therefore, there is a pronounced need for analytical breeding to explore the optimum genetic potential for enhancing yield in soybean [3,4]. 

An analytical breeding strategy, as an alternate breeding approach, requires a better understanding of the factors, or individual traits, responsible for more complex characteristics such as plant growth, development, and yield [5]. This strategy considers secondary traits, which are highly correlated with the trait of interest, as the selection criteria to make empirical selections more efficient toward improving the genetic gain [2,5,6]. The yield potential in soybean is mainly determined by its components, such as the total number of pods, seeds, and nodes per plant, as well as seed size [6,7,8]. Of these traits, the total number of nodes and pods plays a more important role in the final seed yield production [8,9]. Several studies reported a steady increase in the total number of nodes and the total number of pods in soybean cultivars from 1920 to 2010 [2,3,10]. These findings highlight the importance and potential use of the phenotypic and genotypic information in these traits, along with yield per se, as selection criteria in cultivar development programs [10]. The application of analytical approaches to plant-breeding programs has been limited, mainly due to the limited resources available for evaluating several secondary traits that are mostly time- and labor-consuming [5,11]. Most of the analytical breeding studies were conducted on small populations with a limited number of genotypes, and, therefore, the results have limited generalization and limitations in terms of the knowledge of the genome-to-phenome analysis process [9,10,12].

The genetic information of soybean-yield component traits can accelerate the efficiency of cultivar development programs through selecting genotypes with improved genetic gains [13]. Genome-wide association studies (GWASs), as one of the most common genetic approaches, can be implemented on genetically diverse populations to detect the marker-trait associations (MTAs) for soybean-yield components [12]. Up to date, several GWAS approaches, such as mixed linear models (MLM), the multiple loci linear mixed model (MLMM), and fixed and random model circulating probability unification (FarmCPU), have been developed for genetic studies of complex traits [12]. However, due to the narrow genetic base of some plant species, including soybean, these conventional approaches may not have sufficient statistical power to detect reliable MTAs [2,14,15]. Therefore, the development of more sophisticated statistical methods can help to establish effective GWAS methods for plant species with narrow genetic bases.

Machine learning (ML) algorithms, as powerful and reliable mathematical methods, have been considered as an alternative to conventional statistical methods in GWAS analyses [2,16]. Recently, the use of ML algorithms has been reported in different areas such as plant science [14,15,17,18], animal science [19], human science [20], engineering [21], and computer science [22]. The application of ML algorithms in a GWAS was previously investigated in a human-science study by Szymczak, et al. [23], in which different ML algorithms such as artificial neural networks (ANN), Bayesian network analysis (BNA), and random forests (RF) were elucidated for use in GWAS studies focused on human disease studies. One of the most commonly used ML algorithms is RF, developed by Breiman [24], which generates a series of trees from the independent samples and selects the best trees for increasing the prediction performance [25]. The latter algorithm has been widely used in plant genomics [26], phenomics [14], proteomics [27], and metabolomics [28]. The support-vector machine (SVM) is another common algorithm that can detect the behavior and patterns of nonlinear relationships [29,30,31]. Theoretically, SVM should have high performance due to the use of structural risk-minimization, instead of empirical risk-minimization, inductive principles [32]. There are a significant number of reports on the successful use of SVM in prediction problems [19,33,34,35,36]. Support-vector regression (SVR) is known as the regression version of SVM, which is commonly used for continuous variables. There are also reports on the successful use of SVR for addressing plant-prediction problems [37]. 

In this study, we aimed to (1) gain a better understanding of the genetic relationships between soybean yield and its component traits, and (2) investigate the potential use of RF and SVM algorithms in a GWAS for discovering MTAs for soybean-yield components in comparison with the most commonly used conventional GWAS methods. The results of this study may shed light on the potential use of ML algorithms in soybean GWAS studies and may offer new genomic tools for screening high-yielding genotypes with improved genetic gain in large breeding populations. 

## 2. Results

### 2.1. Phenotyping Evaluations

**Figure 1:**
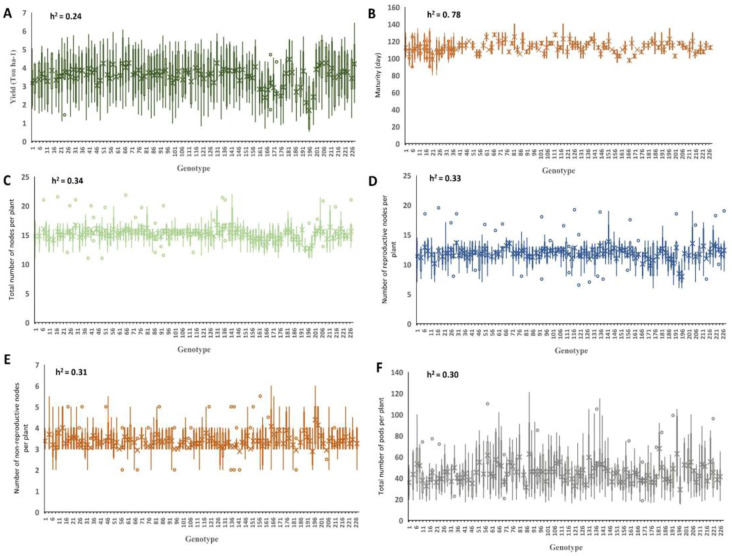
The distribution of seed yield (**A**), maturity (**B**), NP (**C**), NRNP (**D**), RNP (**E**), and PP (**F**) in 227 soybean genotypes across four environments. The estimated heritability is provided for each of the six traits. RNP: the total number of reproductive nodes per plant, NRNP: the total number of non-reproductive nodes per plant, NP: the total nodes per plant, and PP: the total number of pods per plant.

**Figure 2:**
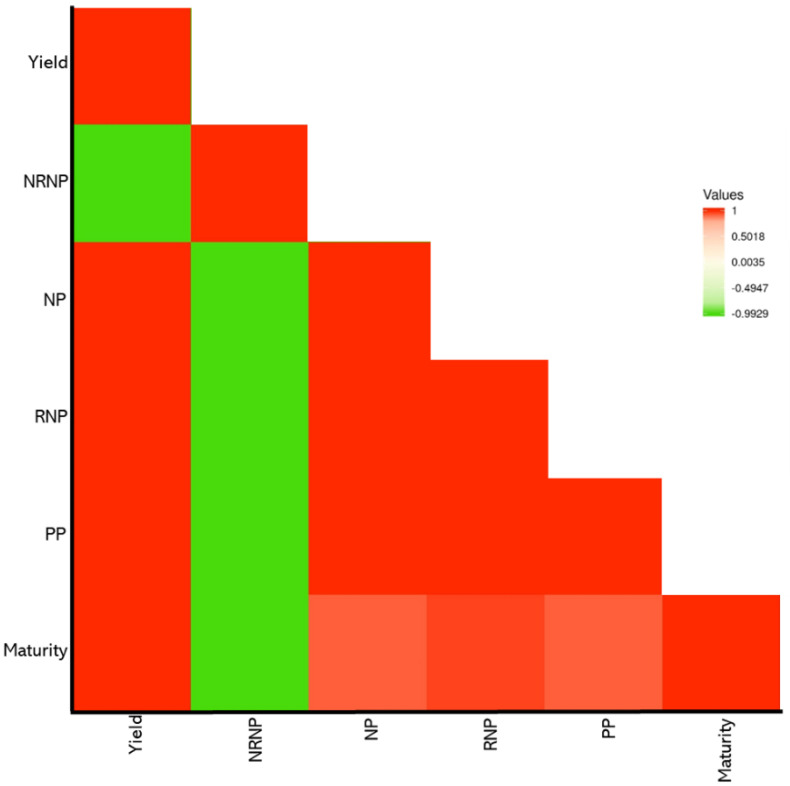
The Pearson correlations among the soybean seed yield, maturity, and yield component traits. RNP: the total number of reproductive nodes per plant, NRNP: the total number of non-reproductive nodes per plant, NP: the total nodes per plant, and PP: the total number of pods per plant. The heat map scale for values is provided by color for the panel.

The panel consisted of 227 soybean genotypes showing different levels of variations among the genotypes for seed yield, maturity, and yield component traits. The distribution of the phenotypic measures for the target traits across the four environments is presented in Figure 1. The highest heritability was observed for maturity (0.78), followed by NP, RNP, NRNP, and PP, with estimated values of 0.34, 0.33, 0.31, and 0.30, respectively (Figure 1). The lowest heritability value of 0.24 was estimated for yield (Figure 1). The average ± standard deviation values for maturity, yield, NP, NRNP, RNP, and PP in the tested GWAS panel were 106 ± 5 days, 3.5 ± 0.45 t ha^−1^, 15.21 ± 0.77 nodes, 3.33 ± 0.28 nodes, 11.89 ± 0.98 nodes, and 45.02 ± 8.54 pods, respectively (Figure S1, see Supplementary Materials). The linear correlations (*r*) among all the measured traits were estimated using the Pearson coefficients of correlation (Figure 2). All the traits were found to be positively correlated with each other, except NRNP, which was negatively associated with yield, maturity, RNP, NP, and PP. NP showed the highest correlation with the RNP (*r* = 0.97) and the NRNP (*r* = −0.63). RNP had the highest correlation with yield (*r* = 0.86) among all the tested yield components (Figure 2).

### 2.2. Population Structure and Kinship

**Figure 3:**
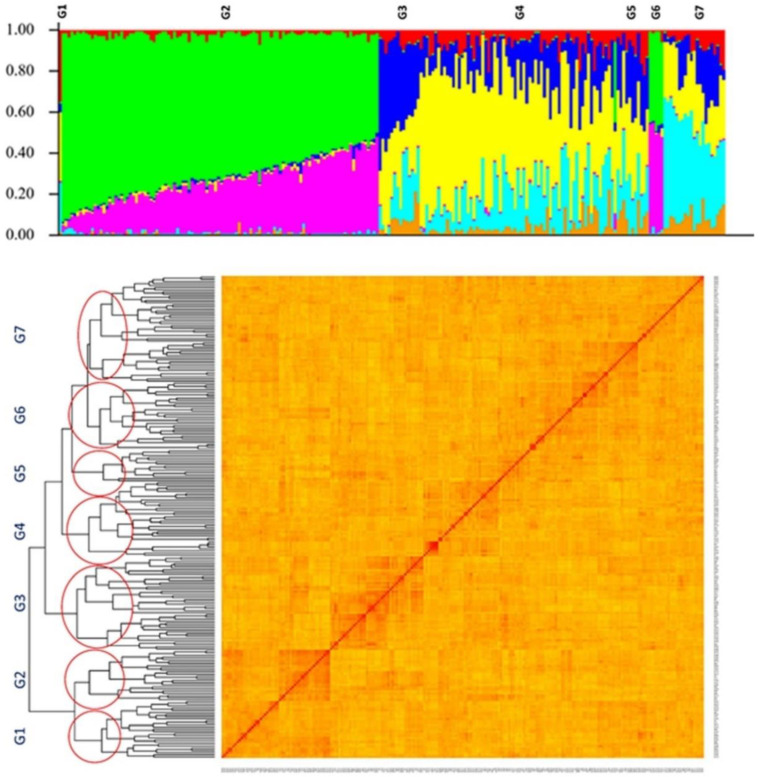
The structure and kinship plots for the 227 soybean genotypes. The x-axis is the number of genotypes used in this GWAS panel, and the y-axis is the membership of each subgroup. G1–G7 stands for the subpopulation.

The structure and kinship profile for the tested population is presented in Figure 3. The result of genotypic evaluations suggested that the tested GWAS panel was composed of four to seven subpopulations. Therefore, we chose to conduct the structure analysis using K = 7 as the appropriate K for the structure profile of the tested GWAS panel (Figure 3). 

### 2.3. GWAS Analysis 

**Figure 4:**
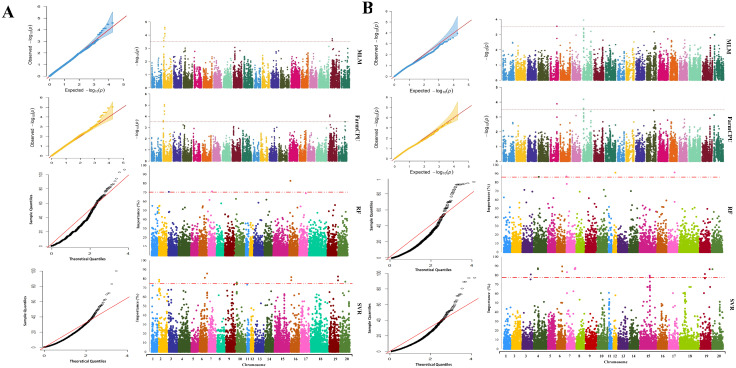
The genome-wide Manhattan and quantile–quantile plots for GWASs of (**A**) maturity and (**B**) seed yield in soybean using MLM, FarmCPU, RF, and SVR methods, from top to bottom, respectively.

The average performance of the tested GWAS methods was compared in Figure S2 (see Supplementary Materials). The association analysis using the MLM method resulted in the identification of nine SNP markers, located on chromosomes 2 and 19, associated with maturity (Table S1). Using FarmCPU resulted in a total of nine maturity-associated SNP markers located on chromosomes 2, 19, and 20 (Figure 4A), of which eight SNPs were also detected by MLM. By using the RF method, a total of three SNP markers on chromosomes 3, 16, and 17 were identified to be associated with this trait, whereas SVR-mediated GWAS detected 12 SNP markers located on chromosomes 2, 6, 10, 16, 19, and 20 (Table S2). For soybean maturity, 3 out of 12 detected MTAs by SVR-mediate GWAS were colocalized with previously reported QTL related to the reproductive period and R8 full maturity (Table 1 and Figure S3). Most of the detected MTAs using MLM and FarmCPU methods were colocalized with previously reported QTL associated with soybean seed weight and Sclerotinia stem rot (Table 1 and Figure S3).

Using the MLM, FarmCPU, RF, and SVR approaches, we identified 2, 3, 5, and 18 SNP markers associated with yield, respectively (Tables S3 and S4). The SNP markers identified by MLM and FarmCPU were located on chromosomes 5 and 8. The markers identified through RF were located on chromosomes 4, 7, 12, and 17. The identified markers using the SVR-mediated GWAS method were located on chromosomes 3, 4, 6, 7, 15, 19, and 20 (Figure 4B). In SVR-mediated GWASs, MTAs were colocalized with eight previously reported yield-related QTL such as seed yield, seed weight, and seed set (Table 2 and Figure S3). However, other tested GWAS methods could not find MTAs colocalized with any previously reported QTL associated with seed yield except for ureide content and water-use efficiency (Table 2 and Figure S3).

**Figure 5:**
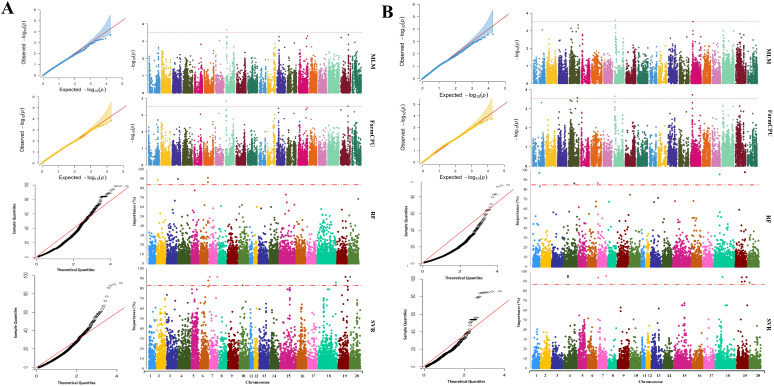
Genome-wide Manhattan and quantile–quantile plots for GWASs of (**A**) the total number of nodes (NP) and (**B**) the total number of non-reproductive nodes (NRNP) in soybean using MLM, FarmCPU, RF, and SVR methods, from top to bottom, respectively.

Using the MLM and FarmCPU methods, we respectively detected one and two SNP markers associated with NP (Table S5). Five and ten SNP markers were associated with NP when RF and SVR methods were used, respectively (Table S6). Most of the MTAs detected by MLM and FarmCPU were colocalized with previously reported QTL related to seed set, seed weight, seed long-chain fatty acid, and pubescence density (Table 3). SVR-mediated GWASs identified MTAs colocalized with three previously reported NP-related QTL (Table 3 and Figure S3). A total of 2, 3, 5, and 10 SNP markers were determined to be associated with NRNP using the MLM, FarmCPU, RF, and SVR methods, respectively (Tables S7 and S8). Chromosome numbers 4, 8, and 15 were identified as carrying SNP markers associated with NRNP using FarmCPU, and the MLM method identified SNP markers located on chromosomes 8 and 15. The detected SNP markers using the SVR method were located on chromosomes 4, 7, 18, 19, and 20, whereas SNP markers identified through RF were located on chromosomes 1, 4, 7, 18, and 19 (Figure 5B). Most of the identified MTAs for NRNP using all GWAS methods were colocalized with previously reported QTL related to seed weight, seed protein, water-use efficiency, first flower, and soybean cyst nematode (Table 4 and Figure S3).

**Figure 6:**
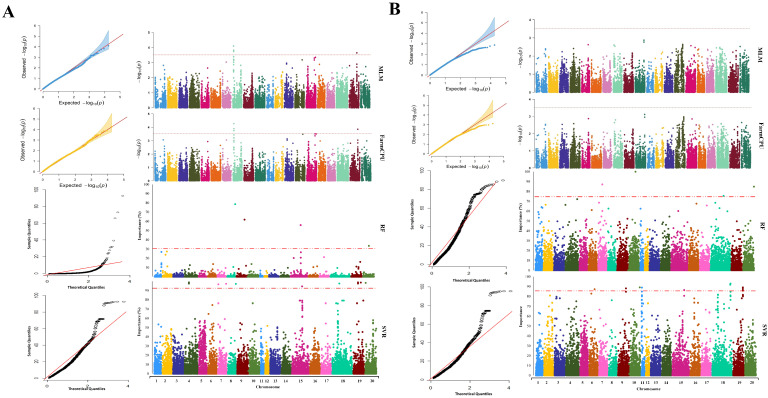
Genome-wide Manhattan and quantile–quantile plots for GWASs of (**A**) the total number of reproductive nodes (RNP) and (**B**) the total number of pods (PP) in soybean using MLM, FarmCPU, RF, and SVR methods, from top to bottom, respectively.

Using the MLM and FarmCPU methods, four SNP markers located on chromosomes 8 and 19 were associated with RNP (Table S9). Using the RF method, four associated SNP markers were identified on chromosomes 8, 9, 15, and 20 (Table S10). Using the SVR method, 11 SNP markers were detected associated with RNP, located on chromosomes 4, 7, 8, 15, 18, 19, and 20 (Figure 6A). Regardless of the type of GWAS methods used in this study, we found SNP markers associated with the trait on chromosome 8. The position of the associated SNP marker on chromosome 8 was identical using all GWAS methods (~450 Kbp). The list of detected QTL for RNP is presented in Table 5 and Figure S3. 

We did not detect any SNP marker associated with PP using the MLM or FarmCPU methods. However, by using the RF method, four SNP markers located on chromosomes 7, 10, 18, and 20 were found to be associated with PP (Table S11). Twelve SNP markers were detected to be associated with PP using SVR. The markers were located on chromosomes 6, 9, 10, 11, 15, 18, and 19 (Figure 6B). The associated SNP markers in chromosome 10 were identified in both RF and SVR with a 4.6 cM distance from each other. Most of the MTAs detected by SVR-mediated GWASs were colocalized with seven previously reported QTL directly related to the pod number (Table 6 and Figure S3). 

### 2.4. Extracting Candidate Genes Undelaying Detected QTL

**Figure 7:**
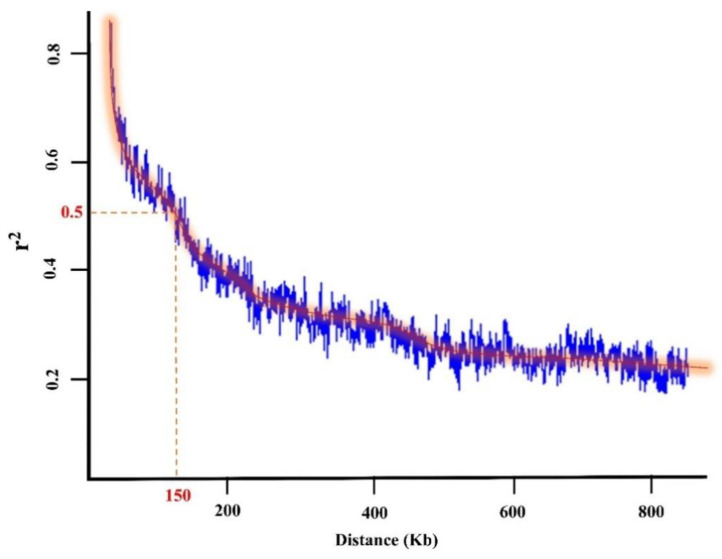
The LD decay distance in the tested 227 soybean genotypes.

**Figure 8:**
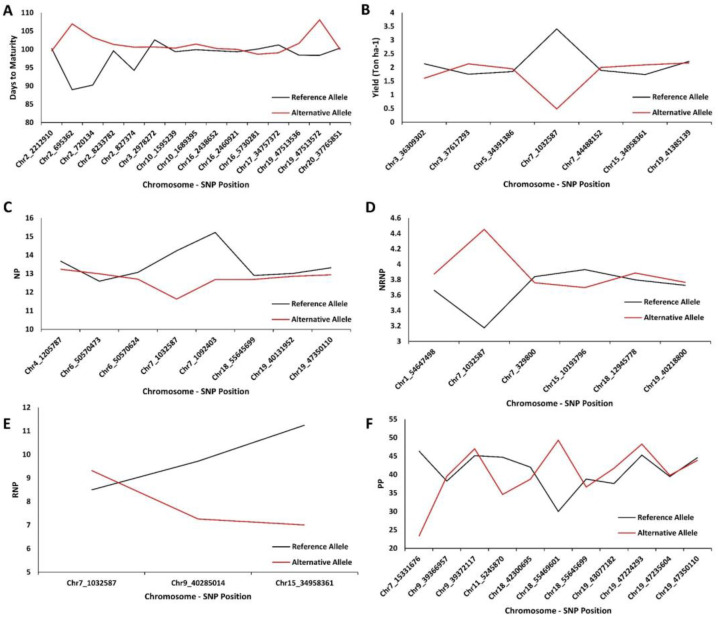
The average effects of the reference allele and the alternative allele from the detected SNP’s peak for maturity (**A**), seed yield (**B**), NP (**C**), NRNP (**D**), RNP (**E**), and PP (**F**) in 227 soybean genotypes across 4 environments. RNP: the total number of reproductive nodes per plant; NRNP: the total number of non-reproductive nodes per plant; NP: the total nodes per plant; and PP: the total number of pods per plant.

To identify the potential candidate genes of each of the detected MTAs, we used the LD decay distance of the panel and selected 150-kbp upstream and downstream of each SNP’s peak as the target regions (Figure 7). The full description of identified candidate genes is presented in Table S12. The effect of each of the identified peak SNPs in explaining the variance of the tested traits is provided in Figure 8. For soybean maturity, three peak SNPs (Chr2_695362, Chr2_720134, and Chr19_47513536) had the highest allelic effects than other detected peak SNPs (Figure 8A). On the basis of the gene annotation and expression within the QTL, *Glyma.02g006500* (GO:0015996) and *Glyma.19g224200* (GO:0010201), which, respectively, encode the chlorophyll catabolic process and phytochrome A (PHYA)-related genes, were identified as the strong candidate genes for maturity. *Glyma.02g006500* (GO:0015996) was exactly detected in the peak SNP position of Chr2_695362, whereas *Glyma.19g224200* (GO:0010201) was 119 Kbp from the detected peak SNP at Chr19_47513536. The yield-related QTL with the peak SNP positioned on Chr7_1032587 had the highest allelic effect compared to other detected peak SNPs (Figure 8B). Within 77 Kbp away from the detected peak SNP (Chr7_1032587), *Glyma.07G014100* (GO:0010817)*,* which encodes the regulation of hormone levels, was identified as the strongest candidate gene in yield. Two peak SNPs, Chr7_1032587 and Chr7_1092403, had the highest allelic effects for the NP trait among all the detected peak SNPs (Figure 8C). In this study, the Chr7_1032587 SNP was associated with yield, NP, and NRNP. The *Glyma.07G205500* (GO:0009693) and *Glyma.08G065300* (GO:0042546) genes, which encode UBP1-associated protein 2C and cell-wall biogenesis, respectively, were detected as plausible genes influencing both NP and NRNP. Both detected candidate genes were collocated at the corresponding peak SNPs at Chr7_1032587 and Chr8_5005929 (Figure 8D). Regarding peak SNPs associated with RNP, the highest allelic effects were found in the peak SNPs of Chr9_40285014 and Chr15_34958361 (Figure 8E). The *Glyma.15G214600* (GO:0009920) and *Glyma.15G214700* (GO:0009910) genes, which encode cell plate formation involved in plant-type cell-wall biogenesis and acetyl-CoA biosynthetic process, respectively, were nominated as strong candidate genes governing NRNP. *Glyma.15G214600* (GO:0009920) and *Glyma.15G214700* (GO:0009910) were 127 and 90 Kbp far from the peak SNP at Chr15_3495836, respectively. For the PP trait, the highest allelic effects were found in peak SNPs at Chr7_15331676, Chr11_5245870, and Chr18_55469601 (Figure 8F). The *Glyma.07G128100* (GO:0009909) gene, which encodes the regulation of flower development, was the strongest candidate gene that can potentially affect PP. *Glyma.07G128100* (GO:0009909) is located in the peak SNP position, Chr7_15331676.

## 3. Discussion

One of the objectives of this study was to attain a better understanding of the roles of soybean-yield component traits in the production of total seed yield and how these traits can be used to facilitate the development of high-yielding soybeans with improved genetic gains. The genetic dissection of soybean-yield components and establishing genetic and genomics toolkits can be used for designing crosses and screening large breeding populations for selecting genotypes with improved yield components [67,68]. The results of this study showed high phenotypic variations for yield and PP across the tested environments, whereas maturity and NP had the lowest phenotypic variations. These findings are in line with the results of previous research studies on yield component traits [2,69], in which high variation for total seed yield and total pods per plant were observed. The heritability and correlation analyses showed that NP had the highest heritability and significant linear correlations with RNP and PP. In addition, PP had the highest correlation with yield among all the tested soybean-yield components. The number of nodes and pods in soybean are known as two of the key soybean-yield components that play important roles in determining the final soybean seed yield [69,70]. Previous studies reported low heritability rates for soybean-yield components, especially NP and PP [2,71], as they are significantly affected by environmental factors [72]. Although, for a given trait, heritability indicates the strength of the relationship between phenotype and genetic variability, it does not necessarily indicate the value of the trait for genetic studies [73]. Different low heritable traits are reported to be highly correlated with significant economic traits [73]. In soybean, for example, yield can be considered as the most important economic trait that is highly determined by its component traits. 

The performance of four GWAS methods was compared in this study, and the results showed that all the methods had acceptable performance in detecting MTAs for the tested traits in this particular population. Among all the tested GWAS methods, SVR-mediated GWASs had a higher aptitude to detect SNP markers with high allelic effects associated with the tested traits in this study. The SVR-mediated GWAS method considers the presence of a nonlinear relationship between input and output variables. This ability is used to build an algorithm with greater prediction accuracies [74]. While conventional GWASs are appropriate approaches for detecting SNP markers with large effects on complex traits, they may not consider a wide range of interconnected biological processes and mechanisms that shape the phenotype of complex traits simultaneously [75]. To discover high-resolution variant-trait associations in ML-mediated GWASs, variable importance values can be used [23]. The variable importance methods based on linear and logistic regressions, support-vector machines, and random forests are well established in the literature [14,76,77,78]. Therefore, MTAs can be discovered by SVR-mediated GWASs as a result of its ability to consider the interaction effects between SNPs rather than the *p*-values for individual SNP-trait GWAS tests. 

In this study, several previously reported QTL were colocalized with identified MTAs using all tested GWAS methods. For maturity, for example, five soybean maturity QTL detected by SVR-mediated GWASs were colocalized with previously reported QTL associated with maturity [39,43]. At the same time, none of the MTAs identified using MLM, FarmCPU, or RF were previously reported to be associated with soybean maturity. Additionally, the peak SNP position of Chr19_47513536 detected by SVR-mediated GWASs had the highest allelic effect among all the detected SNPs for soybean maturity, which is consistent with the findings in Sonah, et al. [39]. For soybean seed yield, SVR-mediated GWAS detected MTAs colocalized with five yield-related QTL [50,55], while none of the detected MTAs using other GWAS methods was previously reported for this trait. We did not find any previous study on the genetic structure of NRNP and RNP, and, therefore, all the identified MTAs in this study are considered as novel genomic regions. For PP, conventional GWAS methods were not able to detect any MTAs. However, SVR-mediated GWASs detected MTAs colocalized with seven QTL related to pod numbers [79]. The average allelic effects of the QTL presented in this study (Figure 8) were estimated using the equation developed by Pimentel, et al. [80]. The RF and SVR-mediated GWAS methods do not specifically measure allele effects, and, therefore, the aim of this study was mostly focused on detecting the MTAs, candidate genes, and QTL underlying the soybean yield, maturity, and yield components. 

Regarding the results of candidate gene identification within identified QTL, several candidate genes were detected using different GWAS methods. For example, among all the detected candidate genes associated with maturity, gene *Glyma.02g006500* (GO:0015996) is a protein ABC transporter 1 that is annotated as a chlorophyll catabolic process and located exactly in the peak SNP position at Chr02_695362. ATP-binding cassette (ABC) transporter genes play conspicuous roles in different plant-growth and developmental stages by transporting different phytochemicals across endoplasmic reticulum (ER) membranes [81]. Because of the central roles played by ABC transporters in transporting biomolecules such as phytohormones, metabolites, and lipids, they play important roles in plant growth, development, and maturity [81,82]. Moreover, recent studies revealed that ER uses fatty acid building blocks made in the chloroplast to synthesize triacylglycerol (TAG). Therefore, ABC transporter genes are important for the normal accumulation of TAG during the seed-filling stage and during maturity [82,83]. Additionally, *Glyma.19g224200* (GO:0010201) in E3 locus, which was previously discovered by Buzzell [84] and molecularly characterized as a phytochrome A (PHYA) gene [85], was detected through the SVR-mediated GWAS. Phytochromes, through PHYTOCHROME INTERACTING FACTOR (PIF), regulate the expression of specific genes encoding rate-limiting catalytic enzymes of different plant growth regulators (e.g., abscisic acid, gibberellins, and auxin) and, therefore, play crucial roles in plant maturity [86]. In addition, PHYB is inactivated after imbibition shade signals, which repress PHYA-dependent signaling in the embryo, which results in the maturing of seeds by preventing germination [87,88]. This is obtained by regulating the balance between abscisic acid and gibberellin. Subsequently, abscisic acid is transported from the endosperm to the embryo by the ABC transporter [88]. 

Among the candidate genes related to NRNP, gene *Glyma.07G205500* (GO:0009693- UBP1-associated protein 2C) that annotated as the ethylene biosynthetic process was located exactly at the peak SNP position at Chr7_37469678. An interaction screen with the heterogeneous nuclear ribonucleoprotein (hnRNP) results in the production of oligouridylatebinding protein 1 (UBP1)-associated protein [89]. It has been well documented that this protein plays an important role in several physiological processes such as responses to abiotic stresses [90], leaf senescence [91], floral development [92], and chromatin modification [93]. In addition, previous studies showed that the production of productive or non-reproductive nodes is completely accompanied by the upregulation or downregulation of this protein [94,95]. In addition, *Glyma.08G065300* (GO:0042546- MADS-box transcription factor), which is associated with cell-wall biogenesis, was located in the SNP position of Chr8_5005929. The genes of the MADS-box family can be considered as the main regulators for cell differentiation and organ determination [96]. The floral organ recognition MADS-box family has been categorized into A, B, C, D, and E classes. Among these classes, class E was shown to be associated with reproductive organ development [97]. Indeed, the activation or repression of this transcription factor leads to the development of nodes to productive or non-productive nodes [98,99,100].

Gene expression dataset developed by Severin, et al. [101] showed that the detected 20 candidate genes for PP using an SVR-mediated GWAS were expressed in flowers, 1 cm pod (7 DAF), pod shell (10–13 DAF), pod shell (14–17 DAF), and seeds. In PP, most of the genes detected by SVR-mediated GWASs are associated with either the auxin influx carrier or auxin response factors (ARFs), gibberellin synthesis, or the response to brassinosteroid [102,103]. Song, et al. [104] and Li, et al. [105] also reported some genes related to PP that were associated with embryo development, stamen development, ovule development, cytokinin biosynthesis, and response gibberellin that we also identified in this study. Soybean seed yield significantly depends on the number of seeds per plant and the seed size [106,107]. These two factors are determined by different factors, from fertilization to seed maturity. Therefore, soybean seed development can be divided into three stages or phases: pre-embryo or seed set, embryo growth or seed growth, and desiccation stages or seed maturation phases [108,109]. In Arabidopsis, a complex signaling pathway and regulatory networks, including sugar and hormonal signaling, transcription factors, and metabolic pathways, have been reported to be involved in seed development [110,111]. Several key genes and transcription factors (e.g., LEAFY COTYLEDON 1 (LEC1), LEC2, FUSCA3 (FUS3), AGAMOUS-LIKE15 (AGL15), ABSCISIC ACID INSENSITIVE 3 (ABI3), YUCCA10 (YUC10), and ARFs) have been determined to control several downstream plant growth regulators pathways to seed development [112,113,114]. Indeed, a high ratio of abscisic acid to gibberellic acid can regulate seed development [115,116]. In soybean, RNA seq analyses for seed set, embryo growth, and early maturation stages of developing seeds in two soybeans with contrasting seed size showed that cell division and growth genes, hormone regulation, transcription factors, and metabolic pathways are involved in seed size and numbers [117].

In general, our results showed that ML-mediated GWAS methods are able to complement the conventional GWAS methods for better identification of the MTAs for traits of interest in soybean. However, the effectiveness of using ML methods in a GWAS should be tested in different soybean populations grown across different environments. In this study, a limited soybean population, which partially covers all the potential genetic variations in the soybean germplasm, was used. Therefore, for further evaluation of the effectiveness of an ML-mediated GWAS, it would be valuable to test the same approaches in a wide range of soybean genotypes using whole-genome sequencing data. In addition, although we used the cross-validation technique and considered several cofactors in our analyses to eliminate the potential false-positive errors, the optimal ML calibrations would be highly recommended to improve capturing the true signals and minimizing the level of errors in ML-based analyses. 

## 4. Materials and Methods

### 4.1. Population and Experimental Design

A panel of 250 soybean genotypes was grown at the University of Guelph, Ridgetown Campus, in two locations, Palmyra (42°25′50.1″ N 81°45′06.9″ W, 195 m above sea level) and Ridgetown (42°27′14.8″ N 81°52′48.0″ W, 200 m above sea level), in ON, Canada, over the course of two years, 2018 and 2019. The randomized complete block designs (RCBD) with two replications were used for all four environments (two locations × two years). In general, there were 500 and 1000 research plots per environment and year, respectively. Each plot consisted of five 4.2 m long rows with 57 seeds per m^2^ seeding rate. The soil type and trials were maintained using standard tillage and cultural practices in both tested locations. No fertilizers were added during the soybean growth and development stages. The herbicides were applied twice before planting and in the middle of the growth period.

### 4.2. Phenotyping

The soybean seed yield (t ha^−1^ at 13% moisture) for each plot was estimated by harvesting three middle rows and adjusted based on the maturity date. Soybean seed yield components, including the total number of reproductive nodes per plant (RNP), the total number of non-reproductive nodes per plant (NRNP), the total nodes per plant (NP), and the total number of pods per plant (PP), were measured using 10 randomly selected plants from each plot. The maturity was recorded as the number of days from planting to physiological maturity (R7) [118] for each genotype.

### 4.3. Genotyping

Young trifoliate leaf tissue for each soybean genotype from the first replication of the trial at Ridgetown in 2018 was collected in a 2 mL screw-cap tube. The leaf samples were freeze-dried for 72 h, using the Savant ModulyoD Thermoquest (Savant Instruments, Holbrook, NY, USA). By using the DNA Extraction Kit (SIGMA^®^, Saint Louis, MO, USA), DNA was extracted for soybean genotypes, and the quantity of DNAs was checked via Qubit^®^ 2.0 fluorometer (Invitrogen, Carlsbad, CA, USA). For genotyping-by-sequencing (GBS), DNA samples were sent to Genomic Analysis Platform at Université Laval (Laval, QC, Canada). The GWAS panel was genotyped via a GBS protocol based on the enzymatic digestion with *ApeKI* [119]. High-quality single-nucleotide polymorphisms (SNPs) were obtained from 210 M single-end Ion Torrent reads that were proceeded with the Fast-GBS.v2 pipeline [120], using the Gmax_275_v2 reference genome. The Markov model was used to impute the missing loci, and SNPs with a minor allele frequency (MAF) less than 0.05 were removed below the threshold. As 23 genotypes did not have sufficient high-quality SNPs, they were eliminated from the experiment. In total, after checking the quality of the reading sequence and removing SNPs with more than 50% heterozygosity, 17,958 SNPs out of 40,712 SNPs were mapped to 20 soybean chromosomes. The minimum number of 403 SNPs was mapped on chromosome 11, and the maximum number of 1780 SNPs was mapped on chromosome 18 (Figure S4). Overall, the average number of SNPs across all the 20 chromosomes was 898, with the mean density of one SNP for every 0.12 cM across the whole genome.

### 4.4. Statistical Analyses

The best linear unbiased prediction (BLUP) as one of the common linear mixed models [121] was used to estimate the genetic values of each soybean genotype. Additionally, the R package *sommer* was used to analyze yield and yield components with ‘environment’ as a fixed effect and ‘genotype’ as a random effect. To control for the possible soil heterogeneity among the plots within a given block and reduce the associated experimental errors, nearest-neighbor analysis (NNA) was used as one of the common error control methods [122,123,124]. Outliers were determined in the raw dataset based on the protocols proposed by Bowley [124] and treated the same as missing data points in the analysis. Overall, the following statistical model (Equation (1)) was used in this study:

(1) $$Y=A_{b}+B_{g}+C_{i}+\varepsilon$$

where *Y* stands for the trait of interest (soybean seed yield and yield component traits); *b* is the vector of block effects that incorporates all the locations and replications, which are added to the overall mean (fixed); *g* is the vector of random genotype effect, in which *g* ~ N(0, σ^2^_g_); *i* is the vector of GxE interaction effects (random), in which *i* ~ N(0, σ^2^_int_); and ε_ij_ stands for the residual effect. *A*, *B*, and *C* stand for the incidence matrices of *b*, *g*, and *i* effects, respectively.

The heritability (Equation (2)) was calculated for soybean seed yield and yield components using the *H2cal* function in the *inti* open-source R package (https://inkaverse.com accessed on 1 May 2022) using the following equation:

(2) $$H^{2}=\frac{\sigma_{G}^{2}\;\;}{\sigma_{G}^{2}+\sigma_{E}^{2}}$$

where $\sigma_{G}^{2}$ stands for the genotypic variance and $\sigma_{E}^{2}$ is the environmental variance. 

### 4.5. Analysis of Population Structure

A total of 17,958 high-quality SNPs from 227 soybean genotypes were used to conduct the population structure analysis using fastSTRUCTURE [125]. Five runs were conducted for K set from 1 and 15 to estimate the most appropriate number of subpopulations by using the K tool from the fastSTRUCTURE software. In order to reduce the confounding, the kinship was also estimated between genotypes of the GWAS panel.

### 4.6. Association Studies

Since different GWAS methods may capture different genomic regions [126], MLM and FarmCPU (the two most common GWAS methods) and RF and SVM (the two most common machine learning algorithms) were used in this study. MLM and FarmCPU were implemented by using the *GAPIT* and *rmvp* packages [127,128], and RF, as well as SVM, were conducted through the *Caret* package [129] in R software version 3.6.1. A brief description of each of the GWAS methods is provided below. 

### 4.7. Mixed Linear Model (MLM) 

This GWAS method is based on the likelihood ratio between the full model, consisting of the marker of interest, and the reduced model, which is known as the model without the marker of interest [130]. MLM is broadly used in GWASs as it effectively corrects inflation from small genetic effects caused by polygenic background and controls the possible bias in the population [130,131,132]. Overall, the equation of MLM would be as follows (Equation (3)):

(3) $$Y=Xa+Z_{MyM}+e_{i}$$

where *Y* is the phenotypic value, *X* is the incident matrix effect, *a* stands for the vector for the incident matrix, *Z_M_* represents the genotype indicator for the Mth SNPs, *yM* is equal to the effect of the SNP_M_ with an assumed normal distribution and mean zero of variance, and *e_i_* represents the residual.

### 4.8. Fixed and Random Model Circulating Probability Unification (FarmCPU) 

This GWAS takes the advantages of using MLM as the random model and stepwise regression as the fixed model iteratively [133]. FarmCPU takes benefits from the random-effect model (REM) for optimizing the SNPs selection based on the p-values (Equation (4)):

(4) $$Y_{i}=U_{i}+e_{i}$$

where *Y_i_* is the observation on the *i*th sample, *e_i_* stands for the residual, and *U_i_* represents the total genetic effect of the *i*th sample.

Additionally, the fixed-effect model (FEM) is used in FarmCPU to test the N number of SNPs simultaneously (Equation (5)):

(5) $$Y_{i}=N_{i1}F_{1}+N_{i2}F_{2}+N_{i3}F_{3}+\cdots +N_{it}F_{t}+M_{ij}K_{j}+e_{i}$$

where *Y_i_* is the observation on the *i*th sample; *N*_*i*1_, *N*_*i*2_, …, *N_it_* represents the genotypes of the t pseudo-QTNs; *F*_1_, *F*_2_, *F*_3_, …, *F_t_* is equal to the corresponding effect for the pseudo-QTNs; *M_ij_* represents the genotype of the *j*th SNPs and *i*th sample; *K_j_* stands for the corresponding effect of the *j*th SNPs; and *e_i_* represents the residual. 

### 4.9. Random Forest (RF)

Random forest (RF) is known as one of the powerful non-parametric regression approaches that is derived from aggregating the bootstrapping in various decision trees [24]. Several decision trees are made based on the training dataset, where the output is the mean of all prediction results from the decision trees (Equation (6)):

(6) $$Y_{i}=\frac{1}{B}\overset{B}{\underset{b=1}{\sum }}T_{b}\;\left(X_{i}\right)$$

where *Y_i_* stands for the predicted value of the genotype *X_i_*, *T* is the total number of constructed trees, and *B* is the total number of samples. In this experiment, a 1000-set of decision trees was constructed in the forest, and the GWAS analysis was conducted by measuring the importance of each feature [134], which was an SNP in this study. 

### 4.10. Support-Vector Regression (SVR) 

Support-vector regression (SVR) is known as one of the common supervised learning methods in prediction problems [135]. This algorithm is based on constructing a set of hyperplanes that can be useful in regression problems [136]. SVR determines the hyperplane by minimizing the difference of squared distances between each datum in the set and its maximum likelihood estimate [137]. In this study, the polynomial kernel was considered in SVR based on the following equation (Equation (7)):

(7) $$L\left(C_{a},C_{b}\right)={\left(a+C_{1}^{T}+C_{2}\right)}^{b}\;\;$$

where $L\left(XC_{a},C_{b}\right)$ represents the polynomial kernel between two data points, *b* is equal to the degree of the kernel, *a* is equal to the constant number, and *T* stands for transpose element.

The association statistics in this algorithm can be achieved by estimating the feature importance that was previously proposed by Weston, et al. [138]. In this experiment, SNP markers were selected as inputs, and the traits were selected as target variables for estimating the feature importance. 

### 4.11. Implementation of ML Algorithms in GWAS

The implementation of ML algorithms in GWASs was reviewed well by Enoma, et al. [139]. In brief, for considering ML algorithms in GWASs, the concept of a GWAS must be seen as a machine learning counterpart. A variable in ML algorithms can be described as genetic information, each possible GWAS covariate as a feature, and phenotypic information as the output or classification, and an individual in the GWAS population can be represented by a single instance of the ML dataset. Additionally, the training, testing, and validation dataset can be considered as the population sample in GWASs.

### 4.12. Variable Importance Measurement

As one of the common indices for tree-based algorithms, the impurity index was chosen as the metric of the feature importance for the RF algorithm. Regarding the SVR algorithm, the variable importance method for SVR [138] was implemented in this dataset. For both algorithms, the importance of each SNP was scaled based on 0 to 100 percent scale. Since there is no confirmed way of defining the significant threshold in the tested algorithms, the global empirical threshold that provides the empirical distribution of the null hypothesis [140,141] was used for establishing the threshold in this study. The global empirical threshold was estimated based on fitting the ML algorithm, storing the highest variable importance, repeating 1000 times, and selecting the SNPs based on α = 0.05. Additionally, the false discovery rate (FDR) is used for setting the threshold both in the FarmCPU and MLM models [142]. To estimate the feature importance in RF and SVR algorithms, a five-fold cross-validation strategy [143] with ten repetitions was applied on the dataset. All of the tested machine learning algorithms were optimized for their parameters for this dataset accordingly.

### 4.13. Extracting Candidate Genes Undelaying Detected QTL

For each tested GWAS model, the flanking regions of each MTA were determined using LD decay distance (Figure 7), and then potential QTL and candidate genes were retrieved using the *G. max* cv. William 82 reference-genome gene models 2.0 in SoyBase (https://www.soybase.org accessed on 1 May 2022). After listing potential candidate genes in defined windows around each significant SNP, at the peak of each QTL, the gene ontology annotation, the GO term enrichment (https://www.soybase.org accessed on 1 May 2022), and the report from previous studies were used as the criteria to select and report the most relevant candidate genes associated with the identified QTL. The Electronic Fluorescent Pictograph (eFP) browser for soybean (www.bar.utoronto.ca accessed on 1 May 2022) was also used to generate additional information such as tissue- and developmental-stage-dependent expression (based on transcriptomic data from Severin, et al. [101]) for the identified candidate genes. A Venn diagram of the MTAs colocalized with previously reported QTL for the tested traits was created using VennPainter software version 1.2.0 [144].

## 5. Conclusions

A better understanding of the genetic architecture of the yield component traits in soybean may enable breeders to establish more efficient selection strategies for developing high-yielding cultivars with improved genetic gains through marker-assisted selections within large breeding populations. Major yield component traits such as maturity, NP, NRNP, RNP, and PP play important roles in determining the overall yield production in soybean. Using correlation and distribution analyses, this study showed the importance of those traits in determining the total soybean seed yield. Furthermore, this study demonstrated the potential benefit of exploiting SVR-mediated GWASs for discovering MTAs associated with yield component traits in soybean. SVR-mediated can be recommended to complement conventional GWAS methods with greater power for detecting MTAs for complex traits such as yield and its components in soybean and possibly other crop species. In order to verify the causal relationship between identified MTAs and the target phenotypic traits, we identified candidate genes within each QTL using gene annotation procedures and information, and the results were promising. Nevertheless, further studies are required to characterize the identified candidate genes in this study and confirm the efficiency of SVR-mediated GWASs for discovering genomic regions with causal relationships with complex traits in plant species.

## Figures and Tables

**Table 1:** The list of MTAs associated with the maturity date, identified using different GWAS methods in this study, which are colocalized with previously reported QTL.

GWAS Method	Chromosome	Peak SNP Position	Co-Located QTL	Reference
MLM	2	2212910	Sclero 3-g31	[38]
		8233782	Seed Weight 6-g1	[39]
FarmCPU	2	2212910	Sclero 3-g31	[38]
		8233766	Seed Weight 6-g1	[39]
	20	37765851	WUE 2-g53	[40]
RF	3	2978272	Leaflet area 1-g2.1	[41]
			Leaflet width 1-g4.1	[41]
			Leaflet area 1-g2.2	[41]
			Leaflet width 1-g4.2	[41]
			Salt tolerance 1-g12	[42]
	16	5730281	Plant height 6-g17	[43]
			Plant height 1-g17	[43]
			First flower 4-g63	[44]
	17	34757372	SDS root retention 1-g6	[45]
SVR	2	695362	Seed linolenic 2-g1	[46]
			Seed linolenic 2-g2	[46]
		720134	SDS 1-g12.1	[47]
			SDS 1-g12.2	[47]
			Ureide content 1-g2	[48]
		827374	SDS 1-g12.3	[47]
	10	1595239	Shoot Cu 1-g8	[49]
		1689395	Seed oil 5-g3	[39]
	16	2438652	Reproductive period 4-g16	[43]
			R8 full maturity 9-g2	[43]
		2460921	Reproductive period 2-g16	[43]
			R8 full maturity 2-g2	[43]
	19	47513536	R8 full maturity 4-g1	[39]
		47513572	First flower 4-g81	[44]

MLM: mixed linear model; FarmCPU: fixed and random model circulating probability unification; RF: random forest; and SVR: support-vector regression.

**Table 2:** The list of MTAs associated with seed yield, identified using different GWAS methods in this study that are colocalized with previously reported QTL.

GWAS Method	Chromosome	Peak SNP Position	Co-Located QTL	Reference
MLM	5	34391386	Ureide content 1-g16.1	[48]
			Ureide content 1-g16.2	[48]
FarmCPU	5	34391386	Ureide content 1-g16.1	[48]
			Ureide content 1-g16.2	[48]
RF	7	1032587	WUE 2-g18	[40]
SVR	3	36309302	First flower 4-g10	[44]
			First flower 3-g2	[50]
			Seed weight 4-g3	[50]
			Seed yield 4-g2	[50]
			R8 full maturity 3-g3	[50]
		37617293	Plant height 3-g17	[51]
			Leaflet shape 1-g1.1	[41]
			Leaflet shape 1-g1.2	[41]
			Leaflet shape 1-g1.3	[41]
			Seed set 1-g32.1	[41]
			Seed set 1-g32.2	[41]
	7	44488152	Seed yield 4-g4	[50]
		1032587	WUE 2-g18	[40]
	15	34958361	SCN 5-g35	[52]
	19	41385139	Seed weight 5-g20	[53]
			Seed weight 4-g18	[50]
			Seed yield 4-g5	[50]
			Shoot Zn 1-g28.1	[49]
			Shoot Zn 1-g28.2	[49]
			Shoot Zn 1-g29.1	[49]
			Shoot Zn 1-g29.2	[49]
			Shoot Zn 1-g29.3	[49]

MLM: mixed linear model; FarmCPU: fixed and random model circulating probability unification; RF: random forest; and SVR: support-vector regression.

**Table 3:** The list of MTAs associated with the total number of nodes per plant (NP), identified using different GWAS methods in this study that are colocalized with previously reported QTL.

GWAS Method	Chromosome	Peak SNP Position	Co-Located QTL	Reference
FarmCPU	19	40131952	Pubescence density 1-g17	[54]
			Seed weight 9-g5.1	[55]
RF	4	1205787	Shoot Ca 1-g10	[49]
	6	50570624	Seed set 1-g51.1	[41]
			Seed set 1-g43.1	[41]
			Seed set 1-g25.1	[41]
			Seed set 1-g43.2	[41]
			Seed set 1-g25.2	[41]
			Seed set 1-g51.2	[41]
		50570473	Seed set 1-g43.3	[41]
			Seed set 1-g51.3	[41]
			Seed set 1-g25.3	[41]
			Pod number 1-g3	[41]
			Seed palmitic 2-g2	[41]
			Seed long-chain faty acid 1-g22	[41]
SVR	6	50570624	Seed set 1-g51.1	[41]
			Seed set 1-g43.1	[41]
			Seed set 1-g25.1	[41]
			Seed set 1-g43.2	[41]
			Seed set 1-g25.2	[41]
			Seed set 1-g51.2	[41]
		50570473	Seed set 1-g43.3	[41]
			Seed set 1-g51.3	[41]
			Seed set 1-g25.3	[41]
			Pod number 1-g3	[41]
			Seed palmitic 2-g2	[41]
			Seed long-chain faty acid 1-g22	[41]
	7	1032587	WUE 2-g18	[40]
		1092403	WUE 2-g18	[40]
			First flower 3-g4	[41]
	18	55645699	Leaflet shape 1-g4.1	[41]
			Leaflet shape 1-g4.2	[41]
			Leaflet shape 1-g4.3	[41]
			Seed stearic 4-g5	[56]
			Node number 1-g6.1	[41]
			Node number 1-g6.2	[41]
			Pod number 1-g1.1	[41]
			Pod number 1-g1.2	[41]
			Pode number 1-g1.3	[41]
			WUE 3-g31	[40]
			Seed weight, SoyNAM 14-g28	[57]
			Lodging, SoyNAM 4-g15	[58]
			Branching 1-g1.1	[41]
			Plant height 5-g4.2	[41]
			Plant height 5-g4.3	[41]
			Shoot p 1-g30	[49]
	19	47350110	Node number 1-g2.3	[41]

MLM: mixed linear model; FarmCPU: fixed and random model circulating probability unification; RF: random forest; and SVR: support-vector regression.

**Table 4:** The list of MTAs associated with the total number of non-reproductive nodes per plant (NRNP), identified using different GWAS methods in this study that are colocalized with previously reported QTL.

GWAS Method	Chromosome	Peak SNP Position	Co-Located QTL	Reference
MLM	15	10193796	Seed protein 6-g2	[59]
			Seed Arg 1-g4	[59]
			Seed coat luster 1-g1.3	[41]
FarmCPU	15	10193796	Seed protein 6-g2	[59]
			Seed Arg 1-g4	[59]
			Seed coat luster 1-g1.3	[41]
RF	1	54647498	First flower 4-g2	[44]
	7	329800	Phytoph 2-g32	[60]
			Phytoph 2-g7	[60]
	18	12945778	SCN 4-g14	[61]
	19	40218800	Seed weight 9-g5.1	[55]
SVR	7	1032587	WUE 2-g18	[40]
	19	40218800	Seed weight 9-g5.1	[55]

MLM: mixed linear model; FarmCPU: fixed and random model circulating probability unification; RF: random forest; and SVR: support-vector regression.

**Table 5:** The list of MTAs associated with the total number of reproductive nodes per plant (RNP), identified using different GWAS methods in this study that are colocalized with previously reported QTL.

GWAS Method	Chromosome	Peak SNP Position	Co-Located QTL	Reference
RF	9	40285014	Shoot Fe 1-g8.1	[49]
			Shoot Fe 1-g8.2	[49]
			Shoot Fe 1-g8.3	[49]
			Shoot Fe 1-g9	[49]
			Shoot Fe 1-g10	[49]
			Shoot Fe 1-g11	[49]
			Soybean mosaic virus 2-g5	[62]
	15	34958361	SCN 5-g35	[52]
SVR	7	1032587	WUE 2-g18	[40]
	15	34958361	SCN 5-g35	[52]

MLM: mixed linear model; FarmCPU: fixed and random model circulating probability unification; RF: random forest; and SVR: support-vector regression.

**Table 6:** The list of MTAs associated with the total number of pods per plant (PP), identified using different GWAS methods in this study that are colocalized with previously reported QTL.

GWAS Method	Chromosome	Peak SNP Position	Co-Located QTL	Reference
RF	7	15331676	Seed weight, SoyNAM 14-g11	[57]
SVR	9	39366957	Pod number 1-g4.1	[41]
			Pod number 1-g4.2	[41]
			Pod number 1-g4.3	[41]
			Seed thickness 2-g4	[41]
	9	39372117	Seed Thr 2-g1	[63]
			Seed Ser 2-g1	[63]
			Seed Tyr 2-g2	[63]
			Seed Lys 2-g2	[63]
			Seed leu 2-g2	[63]
			Seed ile 2-g2	[63]
			Seed Ala 2-g2	[63]
			Seed Gly 2-g2	[63]
	11	5245870	Ureide content 1-g29	[48]
			Pod number 1-g6	[41]
	18	55645699	Leaflet shape 1-g4.1	[41]
		55469601	Leaflet shape 1-g4.2	[41]
			Leaflet shape 1-g4.3	[41]
			Seed stearic 4-g5	[56]
			Node number 1-g6.1	[41]
			Node number 1-g6.2	[41]
			Pode number 1-g1.1	[41]
			Pode number 1-g1.2	[41]
			Pode number 1-g1.3	[41]
			WUE 3-g31	[64]
			Seed weight, SoyNAM 14-g28	[57]
			Lodging, SoyNAM 4-g15	[58]
			Branching 1-g1.1	[41]
			Plant height 5-g4.2	[41]
			Plant height 5-g4.3	[41]
			Shoot p 1-g30	[49]
			Seed yield, SoyNAM 7-g19	[58]
			R8 full maturity, SoyNAM 13-g19	[58]
			Plant height 5-g4.3	[41]
	19	43077182	Seed weight 9-g5.2	[55]
			Seed weight 5-g21	[55]
			First flower 5-g3	[41]
			First flower 5-g17	[41]
		47235604	First flower 4-g77	[44]
			Seed palmitic 1-g19	[65]
		47350110	Leaf carotenoid content 1-g14	[66]
			Ureide content 1-g50.3	[48]
			Ureide content 1-g50.4	[48]
		47224293	Node number 1-g2.3	[41]

MLM: mixed linear model; FarmCPU: fixed and random model circulating probability unification; RF: random forest; and SVR: support-vector regression.

## Data Availability

All datasets will be freely available upon request.

## Supplementary Materials

The following are available online at https://www.mdpi.com/article/10.3390/ijms23105538/s1.
