# Supplementary material for: Machine-Learning-Based Genome-Wide Association Studies for Uncovering QTL Underlying Soybean Yield and Its Components
Source: Int J Mol Sci. 2022 May 16;23(10):5538. doi: 10.3390/ijms23105538 (PMC9141736; doi:10.3390/ijms23105538)
Supplement: Supplementary file 1 [file ijms-23-05538-s001.zip › ijms-1646963 - Supplementary/Supplimentary Materials S1.pdf]

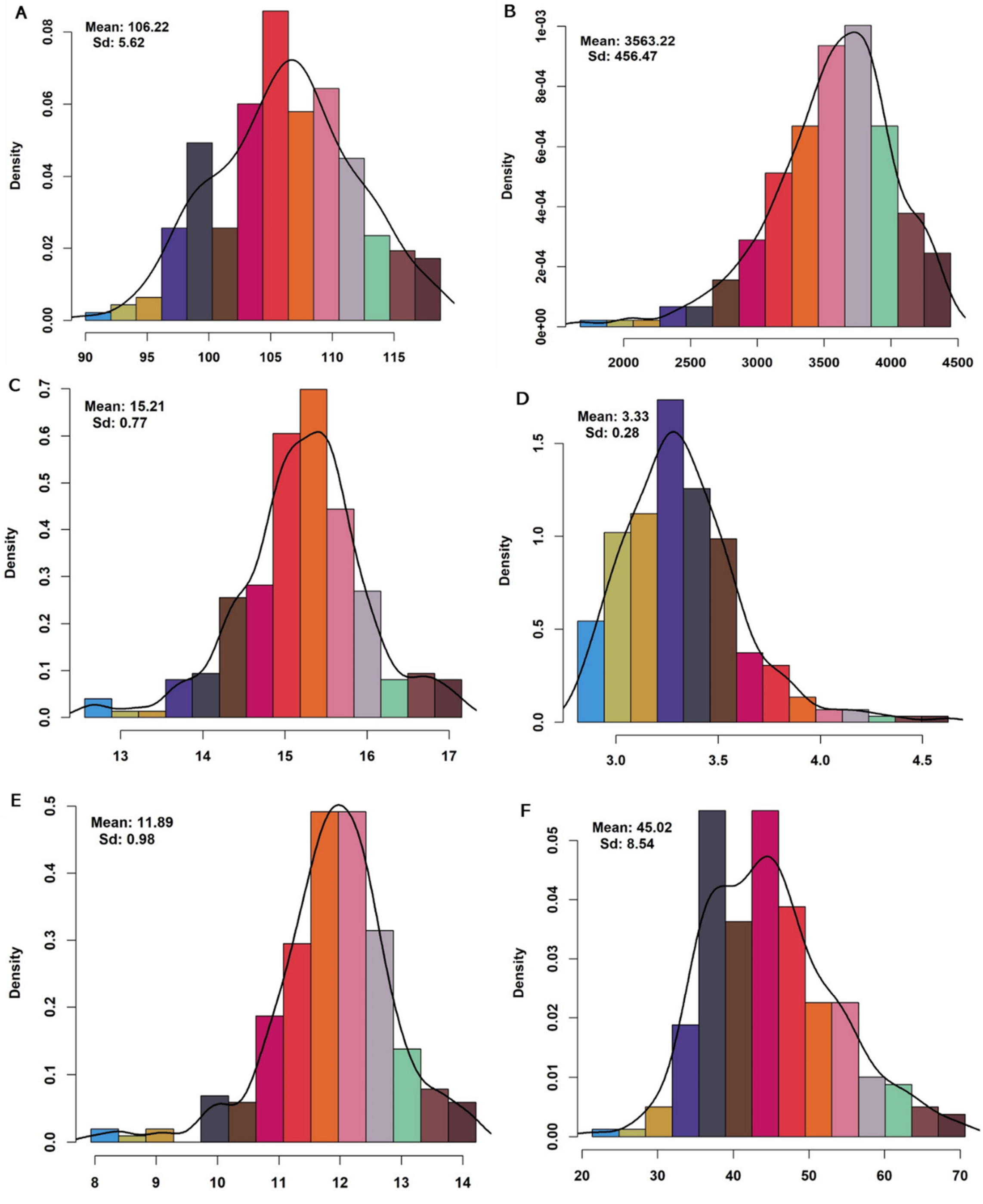

Figure S1. The phenotypic distribution among the soybean (**A**) maturity, (**B**) seed yield, (**C**) The total nodes per plant, (**D**) The total number of non-reproductive nodes per plant, (**E**) Total number of reproductive nodes per plant, and (**F**) The total number of pods per plant.

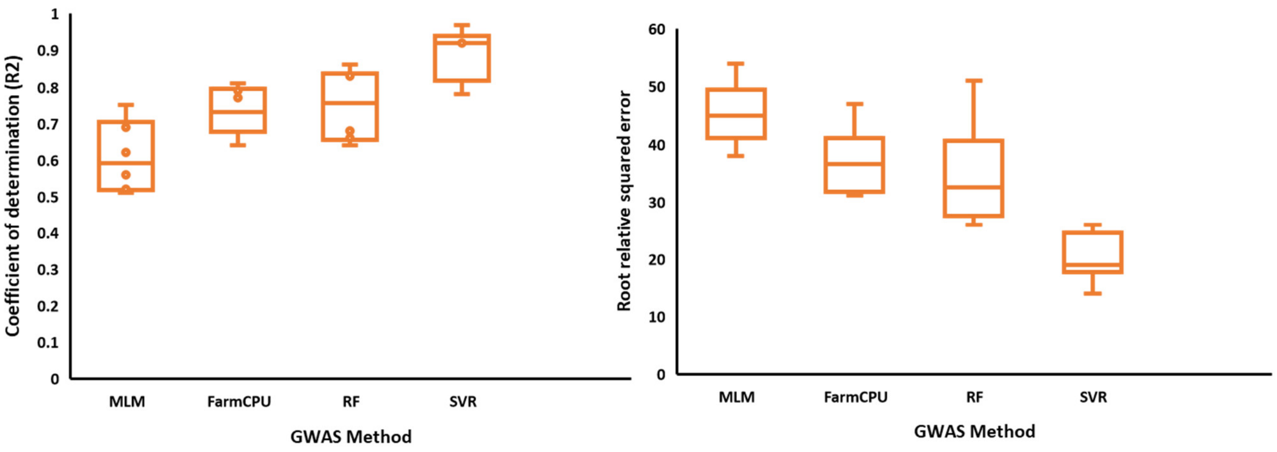

Figure S2. The average coefficient of determination (R^2) and root relative squared error of the tested GWAS algorithms. MLM: Mixed Linear Model, FarmCPU: Fixed and random model circulating probability unification, RF: Random Forest, SVR: Support Vector Regression.

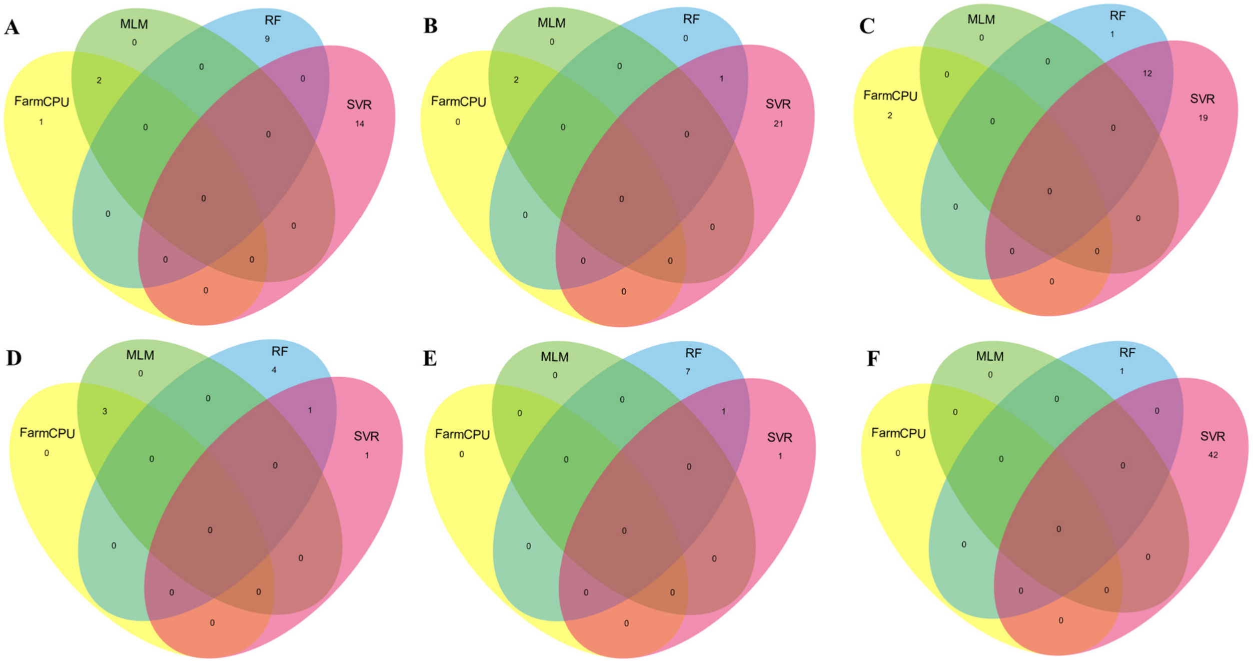

Figure S3. Venn diagram of the MTAs colocalized with previously reported QTL for A) maturity, B) seed yield, C) the total number of nodes (NP), D) the total number of non-reproductive nodes (NRNP), E) The total number of reproductive nodes (RNP), and F) the total number of pods (PP) in soybean using MLM, FarmCPU, RF, and SVR methods.

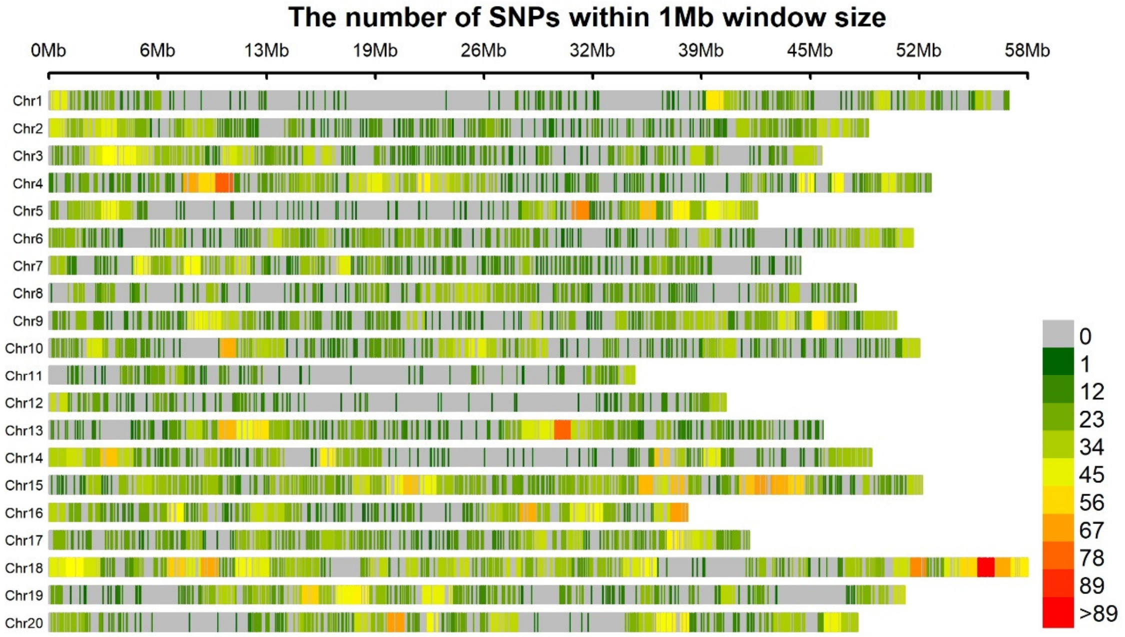

Figure S4. The distribution of SNP markers across the chromosomes of the tested soybean population.

Table S1. The full list of detected MTAs for Maturity using MLM and FarmCPU in the tested soybean population.

GWAS Method	Chromosome	SNP position	Effect	Standard error
MLM	2	2149517	1.792505	0.482867668
	2	2212910	1.762641	0.460495755
	2	8233734	2.927005	0.747248066
	2	8233766	3.10312	0.734120715
	2	8233782	3.139172	0.730754331
	2	8233798	2.926092	0.727978299
	2	8233807	2.933142	0.726771594
	19	9764650	−3.15265	0.849670161
	19	9768960	−3.72545	0.982059376
FarmCPU	2	2212910	1.605745	0.430457289
	2	8233734	2.927764	0.718733406
	2	8233766	3.147648	0.703161392
	2	8233782	3.179466	0.700306634
	2	8233798	2.961624	0.70149717
	2	8233807	2.964826	0.700088857
	19	9764650	−3.01872	0.764478613
	19	9768960	−3.62338	0.900049558
	20	37765851	−1.4674	0.39993614

MLM: Mixed Linear Model, FarmCPU: Fixed and random model circulating probability unification.

Table S2. The full list of detected MTAs for Maturity using RF and SVR in the tested soybean population.

GWAS Method	Chromosome	SNP position	Effect (Scaled)
RF	3	2978272	70.54
	16	5730281	82.82
	17	34757372	68.97
SVR	2	695362	76.83
	2	720134	77.85
	2	827374	78.83
	6	36519422	81.03
	6	50533683	85.63
	10	1595239	74.58
	10	1689395	75.86
	16	2438652	78.28
	16	2460921	81.72
	19	47513536	82.38
	19	47513572	78.19
	20	26583677	76.73

RF: Random Forest, SVR: Support Vector Regression.

Table S3. The full list of detected MTAs for yield using MLM and FarmCPU in the tested soybean population.

GWAS Method	Chromosome	SNP position	Effect	Standard error
MLM	5	34391386	−238.185	64.54998
	8	4816348	−176.858	44.94951
FarmCPU	5	34391386	−239.728	61.54473
	8	4816348	−175.152	43.02462
	8	5005929	−146.799	39.59137

MLM: Mixed Linear Model, FarmCPU: Fixed and random model circulating probability unification.

Table S4. The full list of detected MTAs for yield using RF and SVR in the tested soybean population.

GWAS Method	Chromosome	SNP position	Effect (Scaled)
RF	4	18112413	86.35634
	7	112199	86.752
	7	1032587	78.2727
	12	31320419	91.24594
	17	30546684	91.46945
SVR	3	36309302	75.44121
	3	37617293	80.83272
	4	14417068	87.52
	4	14417041	86.52
	6	28260007	89.53514
	6	28737884	84.21403
	7	44488152	86.83114
	7	37469678	86.65
	7	44171678	87.91655
	7	1032587	83.25
	15	34958361	79.4872
	15	35051401	78.00557
	15	35019563	77.4397
	15	19561019	86.65
	19	41385139	86.47878
	19	18416729	81.254
	19	18394576	76.58
	20	1011439	86.52

RF: Random Forest, SVR: Support Vector Regression.

Table S5. The full list of detected MTAs for the total number of nodes per plant (NP) using MLM and FarmCPU in the tested soybean population.

GWAS Method	Chromosome	SNP position	Effect	Standard error
MLM	8	4816348	−0.28714	0.076246
FarmCPU	8	4816348	−0.2829	0.073228
	19	40131952	0.20595	0.055894

MLM: Mixed Linear Model, FarmCPU: Fixed and random model circulating probability unification.

Table S6. The full list of detected MTAs for the total number of nodes per plant (NP) using RF and SVR in the tested soybean population.

GWAS Method	Chromosome	SNP position	Effect (Scaled)
RF	2	2197493	88.45829
	3	1258563	75.5856
	4	1205787	89.54549
	6	50570624	91
	6	50570473	86.18383
SVR	6	50570624	81.98907
	6	50570473	87.82022
	7	1032587	91.48282
	7	37469678	91.48282
	7	1092403	83.0578
	10	13358967	83.0578
	18	55645699	85.94209
	18	55575915	83.22552
	19	47350110	87.55776
	20	1011439	91.48282

RF: Random Forest, SVR: Support Vector Regression.

Table S7. The full list of detected MTAs for the total number of non-reproductive nodes per plant (NRNP) using MLM and FarmCPU in the tested soybean population.

GWAS Method	Chromosome	SNP position	Effect	Standard error
MLM	8	5005929	0.096527	0.025972
	15	10193796	−0.16419	0.044716
FarmCPU	4	44411975	0.114507	0.030975
	8	5005929	0.093006	0.02481
	15	10193796	−0.15776	0.041557

MLM: Mixed Linear Model, FarmCPU: Fixed and random model circulating probability unification.

Table S8. The full list of detected MTAs for the total number of non-reproductive nodes per plant (NRNP) using RF and SVR in the tested soybean population.

GWAS Method	Chromosome	SNP position	Effect (Scaled)
RF	1	54647498	97.08628
	4	42376190	86.22046
	7	329800	86.52974
	18	12945778	95.54654
	19	40218800	97.80343
SVR	4	14417068	95.35466
	4	14417041	94.35569
	7	37469678	95.54566
	7	1032587	93.5545
	18	19561019	94.34565
	19	40218800	94.07644
	19	18394576	93.95336
	19	30246923	89.76548
	19	18416729	89.34677
	20	1011439	88.54757

RF: Random Forest, SVR: Support Vector Regression.

Table S9. The full list of detected MTAs for the total number of reproductive nodes per plant (RNP) using MLM and FarmCPU in the tested soybean population.

GWAS Method	Chromosome	SNP position	Effect	Standard error
MLM	8	4816348	−0.39027	0.097085
	8	5005929	−0.34216	0.088764
	8	5063716	−0.34861	0.091533
	19	40131952	0.283253	0.075555
FarmCPU	8	4816348	−0.37988	0.092245
	8	5005929	−0.33022	0.08469
	8	5063716	−0.33343	0.087509
	19	40131952	0.273209	0.070496

MLM: Mixed Linear Model, FarmCPU: Fixed and random model circulating probability unification.

Table S10. The full list of detected MTAs for the total number of reproductive nodes per plant (RNP) using RF and SVR in the tested soybean population.

GWAS Method	Chromosome	SNP position	Effect (Scaled)
RF	8	46239110	78.54654
	9	40285014	61.84272
	15	34958361	55.89821
	20	26567069	33.38569
SVR	4	14417041	98.76576
	4	14417068	97.43655
	7	37469678	97.23576
	7	1032587	96.65465
	8	46239110	97.14433
	15	34958361	94.44051
	18	19561019	97.54655
	19	30246923	98.54655
	19	18416729	98.75676
	19	18394576	97.54578
	20	1011439	98.23478

RF: Random Forest, SVR: Support Vector Regression.

Table S11. The full list of detected MTAs for the total number of pods per plant (PP) using RF and SVR in the tested soybean population.

GWAS Method	Chromosome	SNP position	Effect (Scaled)
RF	7	15331676	87.0475
	10	27244270	100
	18	42300695	75.49254
	20	47642298	84.81901
SVR	6	28737884	87.21842
	9	39366957	88.02327
	9	39372117	88.02327
	10	49302884	89.10978
	11	5245870	88.83899
	15	37810860	86.3949
	18	55645699	92.90795
	18	55469601	91.78526
	19	47235604	86.00507
	19	47350110	85.79695
	19	47224293	87.52014
	19	43077182	89.04024

RF: Random Forest, SVR: Support Vector Regression.
